# Endogenous piRNAs Can Interact with the Omicron Variant of the SARS-CoV-2 Genome

**DOI:** 10.3390/cimb45040193

**Published:** 2023-04-03

**Authors:** Aizhan Rakhmetullina, Aigul Akimniyazova, Togzhan Niyazova, Anna Pyrkova, Saltanat Kamenova, Aida Kondybayeva, Alma-Gul Ryskulova, Anatoliy Ivashchenko, Piotr Zielenkiewicz

**Affiliations:** 1 Institute of Biochemistry and Biophysics, Polish Academy of Sciences, 02-106 Warsaw, Poland; 2 Department of Technology of Production of Livestock Products, A. Baitursynov Kostanay Regional University, Kostanay 110000, Kazakhstan; 3 Higher School of Medicine, Faculty of Medicine and Healthcare, Al-Farabi Kazakh National University, Almaty 050040, Kazakhstan; 4 Faculty of Biology and Biotechnology, Al-Farabi Kazakh National University, Almaty 050040, Kazakhstan; 5 Center for Bioinformatics and Nanomedicine, Almaty 050060, Kazakhstan; 6 Department of Population Health and Social Sciences, Kazakhstan’s Medical University “KSPH”, Almaty 050060, Kazakhstan

**Keywords:** Omicron, SARS-CoV-2, piRNAs, coronavirus genome, translation, replication

## Abstract

Severe acute respiratory syndrome coronavirus 2 (SARS-CoV-2), which caused the COVID-19 pandemic, can still infect populations in many countries around the globe. The Omicron strain is the most mutated variant of SARS-CoV-2. The high transmissibility of the strain and its ability to evade immunity necessitate a priority study of its properties in order to quickly create effective means of preventing its spread. The current research aimed to examine the in silico interaction between PIWI-interacting RNAs (piRNAs) and the SARS-CoV-2 genome (gRNA) to identify endogenous piRNAs and propose synthetic piRNAs with strong antiviral activity for drug development. This study used validated bioinformatic approaches regarding the interaction of more than eight million piRNAs with the SARS-CoV-2 genome. The piRNAs’ binding sites (BSs) in the 5′UTR were located with overlapping nucleotide sequences termed clusters of BSs. Several BSs clusters have been found in the nsp3, nsp7, RNA-dependent RNA polymerase, endoRNAse, S surface glycoprotein, ORF7a, and nucleocapsid. Sixteen synthetic piRNAs that interact with gRNA have been proposed with free binding energy ranging from −170 kJ/mol to −175 kJ/mol, which can be used to create drugs that suppress the reproduction of SARS-CoV-2.

## 1. Introduction

Severe acute respiratory syndrome coronavirus 2 (SARS-CoV-2), which caused the COVID-19 pandemic, is still capable of infecting populations in many countries around the globe. SARS-CoV-2 causes disease in humans and many vertebrates [1], which facilitates the study of the pathogenic effect of SARS-CoV-2 and the testing of drugs intended for humans. In addition to typical fever and respiratory symptoms, many patients with COVID-19 suffer from a variety of neurological complications and cancer [2,3,4,5]. SARS-CoV-2 is distributed in many strains: B.1.1.7 (Alpha), B.1.351 (Beta), B.1.525 (Eta), B.1.427/B.1.429 (Epsilon), B.1.526 (Iota), B.1.617.1 (Kappa), B.1.617.2 (Delta), C.37 (Lambda), P.1 (Gamma), P.2 (Zeta), P.3 (Theta) and the newly discovered B.1.1.529 (Omicron) [6], which requires their features to be established and the requirement for specific treatment methods. As an object of research, we chose the Omicron strain (B.1.1.529), the most mutated variant of SARS-CoV-2. Its high transmissibility and ability to evade immunity are of significant concern. 

Due to the absence of effective treatments for coronavirus strains, medications must be created that can both treat and stop the spread of infection. One of the properties of the coronavirus genome (gRNA) that contributes to its survival in animals lies in the structure of gRNA being similar to the mRNA structure of protein-coding genes. The gRNA nucleotide sequence contains 5′-untranslated region (5′UTR), protein-coding region (CDS), and 3′-untranslated region (3′UTR), which allows it to be similar to the mRNA structure of many genes, including being tens of thousands of nucleotides in length. A number of studies have examined the features of all of the functional regions of gRNA that could serve as the basis for the development of methods to prevent SARS-CoV-2. Since gRNA contains 5′UTR, there have been attempts to elucidate the role of this region of SARS-CoV-2 in the pathogenicity of the coronavirus. The viral 5′UTRs are believed to provide reliable expression of viral mRNAs. The above features can be used as potential therapeutic targets [7]. The 5′UTR segment of coronavirus genomes performs important functions in the viral replication cycle and viral gRNA translation [8]. Several studies have shown that miRNA binding sites (BSs) are located in the 5′UTR, which can interfere with protein synthesis [9,10,11]. The effect of compounds on the 5′UTR of SARS-CoV-2 acting on the 5′UTR mRNAs of human protein-coding genes has been studied [12,13,14,15]. The authors concluded that the combined non-structural protein 1 (NSP1) degradation of spliced mRNAs and the repression of translation of single-exon genes, along with the unique characteristics present in the viral 5′UTRs, provide robust expression of viral mRNAs. [15]. The effect of the 5′UTR gRNA structure as a potential therapeutic target against SARS-CoV-2 has been studied [16,17,18,19]. The spike protein (S protein) encoded in gRNA has attracted the interest of researchers since it is thought to play a vital role in viral penetration into the recipient cell [6,20,21,22,23,24]. Several publications have elucidated the role of the S protein as a target for viral suppression [1,22,25,26,27]. Based on a number of studies, individual protein-coding regions of gRNA can be utilized autonomously to examine the effect of the PIWI-interacting RNAs(piRNAs) we identified that act within the *NSP3* gene [28,29,30]. Regions encoding Nsp2, Nsp3, S1, ORF8 [31], and the omicron S protein [32] have been studied. The S protein, receptor-binding domain (RBD), nucleocapsid (N), orf3a, orf8, nsp3, nsp13, and membrane (M) antigenic specificities have been examined [33]. The expression and subcellular localization of 11 distinct SARS-CoV-2 nonstructural replicase proteins were systematically analyzed [34]. The development of antiviral strategies requires an understanding of the SARS-CoV-2 replication and transcription processes. The replicase polyprotein is essential for viral reproduction [35].Papain-like protease is a highly conserved proteolytic enzyme that cleaves the peptide bond between Nsp1, Nsp2, Nsp3, and Nsp4. As a valid therapeutic targets, it inhibits viral replication and boosts host immunity [36]. Surprisingly, free Hb at 1mM inhibited viral replication (99%), and its interaction with SARS-CoV-2 was localized in the RBD region of the S protein. The authors in this study identified that five proteins (S, N, M, Nsp3, and Nsp7) of SARS-CoV-2 recruit Hb/metabolites [37]. The above analysis of the properties of gRNA regions shows that the 5′UTR and each of the protein-coding regions can be modified by various agents. However, for the most part, these agents are difficult to use to suppress the viability of the coronavirus in vivo. piRNAs were first discovered more than 15 years ago [38], but they were not used as direct regulators of the protein translation process on mRNA due to a number of misconceptions. It has been shown that piRNA-containing exosomes/microvesicles release them to manifest antiviral immunity. However, it remains unknown whether piRNAs can target SARS-CoV-2 and whether the PIWI-piRNA system is crucial for antiviral actions [39,40]. Recently created databases of piRNAs contain data on more than eight million of these molecules [41,42]. In recent years, the interaction of piRNAs with human gene mRNAs has been established [43,44]. It has been shown that piRNAs can interact with the gRNA of the Delta strain [45]. This information allowed us to suggest that piRNAs can influence the expression of protein-coding genes and participate in the suppression of SARS-CoV-2 reproduction in the human body. It must be borne in mind that synthetic piRNAs (spiRNAs) created at random, in addition to suppressing the reproduction of the virus, can suppress the expression of human genes, causing side effects. For this reason, numerous successful experiments on the suppression of coronaviruses in cell cultures and in experimental animals have not been brought to clinical use. This research focuses on the in silico analysis of the piRNA interactions with the genome of the SARS-CoV-2 Omicron strain in order to determine endogenous piRNAs and suggest spiRNAs for drug development. To elucidate this problem, we identified endogenous piRNAs capable of suppressing viral replication and based on these piRNAs, propose spiRNAs.

## 2. Materials and Methods

The nucleotide (nt) sequence of the Omicron variant (B.1.1.529) of the SARS-CoV-2 gRNA has been obtained from the National Center for Biotechnology Information (NCBI) (https://www.ncbi.nlm.nih.gov/, accessed on 17 January 2022). From Wang et al., 8.426 million piRNAs’ nucleotide sequences were taken [41].

The BSs of piRNAs in SARS-CoV-2 gRNA were predicted using the MirTarget program [46]. This program predicts the following features of piRNA-gRNA binding: (a) initiation of piRNA-gRNA binding from the first nucleotide of the gRNA; (b) localization of piRNA BSs in the 5′UTR, CDS and 3′UTR of gRNA; (c) nucleotide interaction schemes between piRNA and gRNA; (d) the free energy of interaction between piRNA and gRNA (ΔG, kJ/mol). The ratio ΔG/ΔGm (%) is determined for each BS (ΔGm equals the free energy of binding of piRNA to an entirely complementary canonical nucleotide sequence). From the calculated data, only the piRNAs whose nucleotides interacted with the gRNA via canonical (G-C and A-U) and non-canonical (G-U and A-C) nucleotides with a certain ΔG value were selected. MirTarget finds hydrogen bonds between adenine (A) and uracil (U), guanine (G) and cytosine (C). The free energy (ΔG) of the G and C pair interaction is 6.37 kJ/mol, the A and U pair is 4.25 kJ/mol, G and U, A and C are 2.12 kJ/mol. The distance between A and C (1.04 nm) and G and U (1.02 nm) bonds is similar to the distance between A and U, G and C, which is 1.03 nm [47,48,49,50]. The number of hydrogen bonds in the G-C, A-U, G-U, and A-C interactions is 3, 2, 1, and 1, respectively. MirTarget differs from other programs in terms of finding the piRNA to gRNA in that it accounts for piRNA-gRNA interactions over the entire piRNA sequence; it considers non-canonical G-U and A-C pairs; and calculates the free energy of piRNA-gRNA interactions. 

**Figure 1:**
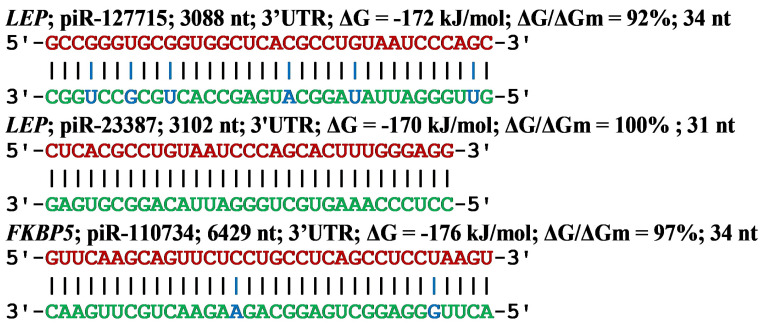
Nucleotide sequences of piRNA BSs in the 3′UTR of mRNA and nucleotide sequences of piRNAs. Note: The nucleotides of piRNAs forming canonical and non-canonical pairs with mRNA are highlighted in green and blue, respectively.

As an example of the interaction of piRNAs with the mRNA *LEP* gene, consider the diagram of piRNAs binding to the mRNA site in Figure 1 from the article [44]. Of the 34 pairs of nucleotides, 6 pairs are non-canonical (five G-U and one A-C). The distance between the nucleotides in pairs varies from 1.02 nm (G-U) to 1.04 nm (A-C). This piR-127715 complex with the mRNA *LEP* gene forms a helix almost identical to the helix containing only canonical pairs of nucleotides. Below is a scheme of the fully complementary interaction of all piR-23387 nucleotides with the mRNA *LEP* gene. The piR-110734 complex with the mRNA *FKBP5* gene forms a secondary and tertiary structure almost identical to the secondary and tertiary structures when only canonical nucleotide pairs are used.

## 3. Results

**Figure 2:**
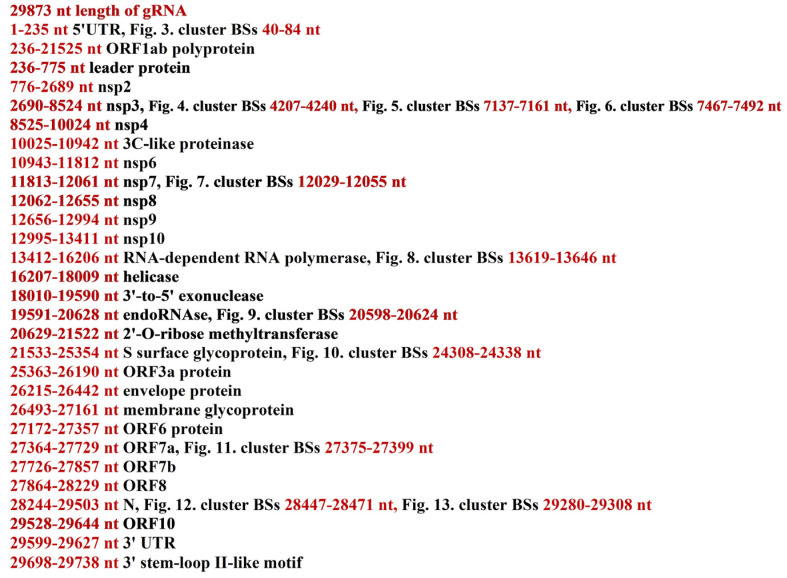
Localization of piRNA BSs clusters in the gRNA of the Omicron variant of SARS-CoV-2.

**Figure 3:**
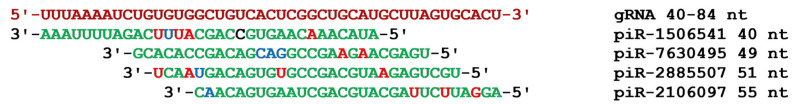
Nucleotide sequences of four piRNAs and the gRNA BS in a region from 40 nt to 84 nt. Note: The piRNA nucleotides that form canonical pairs with gRNA are highlighted in green and non-canonical pairs are highlighted in blue. The piRNA nucleotides that do not interact with gRNA are highlighted in red. The names of the piRNAs are followed by the beginning of their BSs.

The first region of the gRNA nucleotide sequence that interacted with piRNAs was located at the 5′UTR (Figure 2). The BSs of four piRNAs form a cluster with a partial overlap of nucleotides (Figure 3). 

The overlap of the nucleotide sequences of piRNA BSs in gRNA leads to the competition of different piRNAs while suppressing the start of the process of translation of coronavirus proteins. Given that the genes encoding these piRNAs can be located in different parts of the human genome, in the case of mutations that disrupt the synthesis of one of these piRNAs, the body will retain the ability to defend itself against the coronavirus. In addition to the above four piRNAs interacting with gRNA with free energy from −130 kJ/mol and higher, in the 5′UTR BSs cluster at position 156 nt, piR-2388809 also bound with a ΔG value of −132 kJ/mol (Table S1). Therefore, by synthesizing endogenous piRNAs, a human acquires the ability to protect themself from a deadly pathogen.

**Figure 4:**
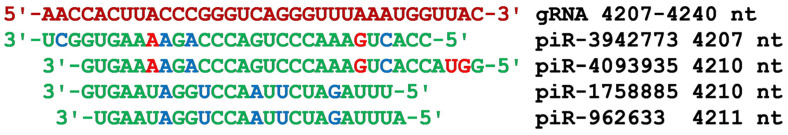
Nucleotide sequences of the piRNA BSs cluster in the CDS of gRNA from 4207 nt to 4241 nt and the nucleotide sequences of four piRNAs. Note: The piRNA nucleotides that form canonical pairs with gRNA are highlighted in green and non-canonical pairs are highlighted in blue. The piRNA nucleotides that do not interact with gRNA are highlighted in red. The names of the piRNAs are followed by the beginning of their BSs.

In the nsp3 protein-coding part of gRNA, we identified several clusters of BSs for four or more piRNAs (Figure 2). Consider the properties of these clusters of BSs in the order of their location in gRNA. In the CDS region of gRNA from 4207 nt to 4241 nt, the BSs of four piRNAs were located (Figure 4). The BSs of these piRNAs were located with a partial overlap of nucleotides, that is, they formed a cluster of BSs. piR-3942773 and piR-4093935 interacted with gRNA with ΔG values of −138 kJ/mol and −136 kJ/mol (Table S1). Such free energy of interaction allows these piRNAs to be strongly attached to nsp3 gRNA and inhibit protein synthesis. The BSs cluster contained the ACUU tetranucleotide, which ensures the binding of the UGAA tetranucleotide of piRNAs using canonical base pairs. In the center of piR-3942773 and piR-4093935 was the CCCAGUCCCAAA oligonucleotide, which also bound to gRNA via canonical GGGUCAGGGUUU base pairs. In piR-1758885 and piR-962633, the UGAAUAGGUCCAAUUCUAGAUUU polynucleotide provided complementary interaction with gRNA through canonical and non-canonical base pairs along almost their entire length.

**Figure 5:**
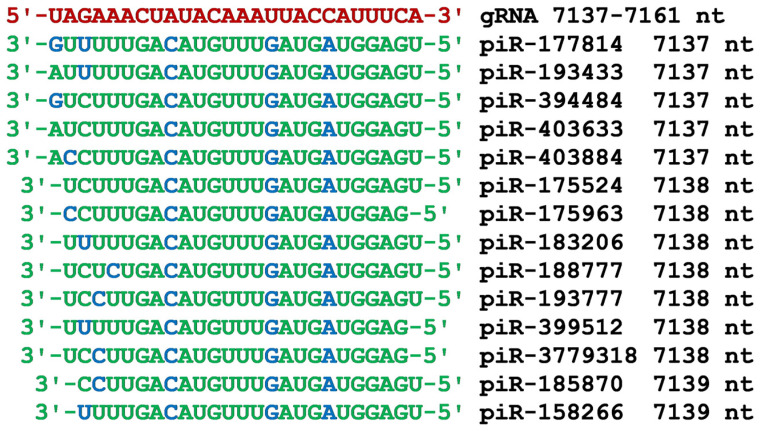
Nucleotide sequences of the piRNA BSs cluster in the CDS of gRNA from 7137 nt to 7161 nt and the nucleotide sequences of four piRNAs. Note: The piRNA nucleotides that form canonical pairs with gRNA are highlighted in green and non-canonical pairs are highlighted in blue. The names of the piRNAs are followed by the beginning of their BSs.

The third cluster of piRNA BSs from 7137 nt to 7161 nt was also located in the nsp3 protein-coding region of gRNA (Figure 5). A feature of this cluster is the presence of a large number of piRNA BSs, all nucleotides of which fully complementary interacted with gRNA. Such a set of piRNAs is guaranteed to provide high human protection against the coronavirus. Only a significant mutation in the cluster of gRNA BSs could increase the protection against the coronavirus of these endogenous piRNAs. Except for piR-193777, the remaining 13 piRNAs had the same oligonucleotide UUGACAUGUUUGAUGAUGGAG, which provides strong interaction with gRNA mainly due to canonical base pairs. The first four GGAG nucleotides from the 5′-end of piRNAs provided strong binding to gRNA through G-C pairs with three hydrogen bonds. The example of this BSs cluster shows that the human genome, in the process of evolution, created protection against SARS-CoV-2. It seems that it is better for the human genome to maintain a constant set of piRNAs than to frequently adapt to coronavirus mutations, although both ways of protecting against the coronavirus take place. Further research on different strains of SARS-CoV-2 and other coronaviruses can be conducted to see whether this claim is true.

**Figure 6:**
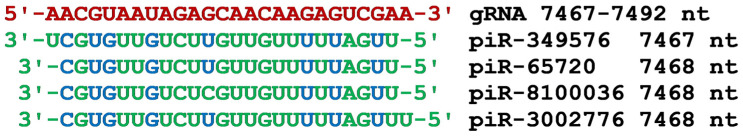
Nucleotide sequences of the four piRNA BSs cluster in the region from 7467 nt to 7492 nt of gRNA and the nucleotide sequences of piRNAs. Note: The piRNA nucleotides that form canonical pairs with gRNA are highlighted in green and non-canonical pairs are highlighted in blue. The names of the piRNAs are followed by the beginning of their BSs.

The BSs cluster of four piRNAs with gRNA is shown in Figure 6. All piRNAs interacted with gRNA through canonical and non-canonical base pairs. The nucleotide sequence of piR-65720 CGUGUUGUCUUGUUUUUUAGUU was identical to part of the nucleotide sequences of other piRNAs, except for the replacement of the U nucleotide with the C nucleotide at position 7478 of piR-8100036 (Figure 6). As a result, we suggest that the identified four piRNAs may significantly suppress the synthesis of the protein encoded by the nsp3 region.

**Figure 7:**
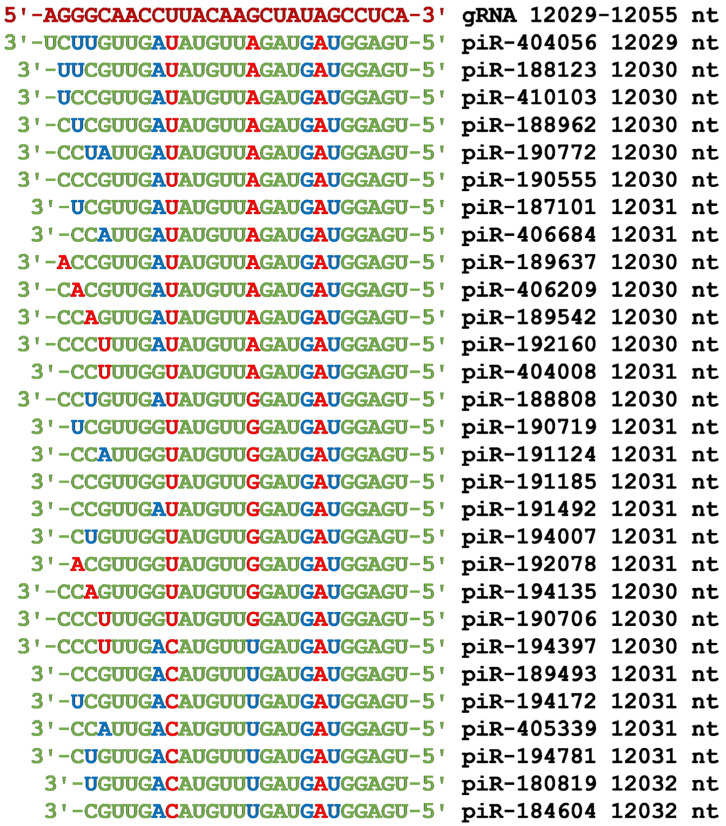
Nucleotide sequences of the 29 piRNA BSs cluster in the CDS of gRNA from 12,029 nt to 12,055 nt and the nucleotide sequences of piRNAs. Note: The piRNA nucleotides that form canonical pairs with gRNA are highlighted in green and non-canonical pairs are highlighted in blue. The piRNA nucleotides that do not interact with gRNA are highlighted in red. The names of the piRNAs are followed by the beginning of their BSs.

The largest number of piRNAs bound in the BSs cluster was located from 12,029 nt to 12,055 nt in the nsp7 protein-coding region of gRNA (Figure 2). The nucleotide sequences of the BSs of 29 piRNAs were arranged so that the 5′ end of the piRNAs binds at the same gRNA position (12,055 nt) despite their different binding positions from the 3′ end (Figure 7). This localization of BSs for all 29 piRNAs is not random and was due to the identity of the UGGAGU oligonucleotide of the 5′-end of piRNAs, which bound to gRNA only through canonical base pairs. Other conserved nucleotides in all piRNAs were GAUG and AUGUU, which also bound to gRNA with the formation of canonical base pairs.

**Figure 8:**
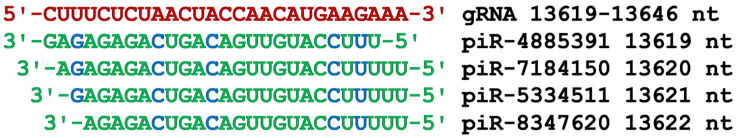
Nucleotide sequences of the four piRNA BSs cluster in the region of gRNA from 13,919 nt to 13,646 nt and the nucleotide sequences of piRNAs. Note: The piRNA nucleotides that form canonical pairs with gRNA are highlighted in green and non-canonical pairs are highlighted in blue. The names of the piRNAs are followed by the beginning of their BSs.

All nucleotides of four piRNAs bound to gRNA to form canonical and non-canonical pairs, as shown in Figure 8. The BSs of these piRNAs formed a cluster from 13,919 nt to 13,646 nt in the gRNA encoding RNA-dependent RNA polymerase (Figure 2). The AGAGACUGACAGUUGUACCUUU oligonucleotide site was shared by the four piRNAs, and termination of synthesis of any of the four piRNAs could be replaced by the remaining three piRNAs.

**Figure 9:**
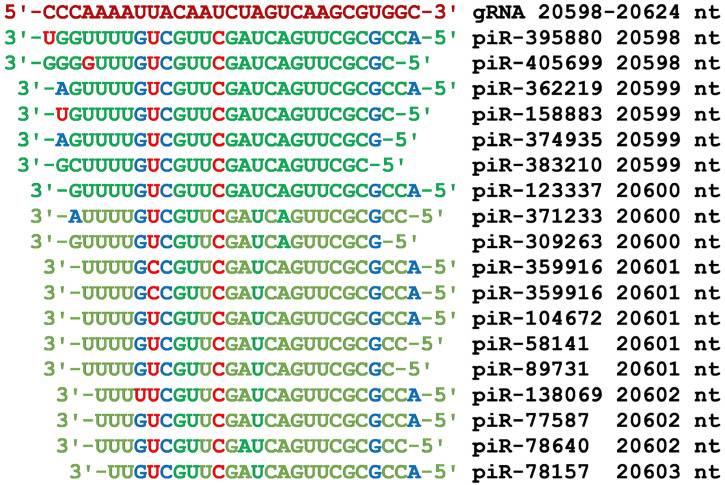
Nucleotide sequences of the piRNA BSs cluster in the region of gRNA from 20,598 nt to 20,624 nt and the nucleotide sequences of piRNAs. Note: The piRNA nucleotides that form canonical pairs with gRNA are highlighted in green and non-canonical pairs are highlighted in blue. The piRNA nucleotides that do not interact with gRNA are highlighted in red. The names of the piRNAs are followed by the beginning of their BSs.

In the region from 20,598 nt to 20,624 nt of gRNA, there was a BSs cluster of eighteen piRNAs that inhibit endoRNAase synthesis (Figure 9). All these piRNAs contained the GAUCAGUUCGC oligonucleotide, which interacted with gRNA only through canonical base pairs. All the piRNAs had the same UCGUUC hexanucleotide flanked by nucleotides that do not bind gRNA (Figure 9). 

**Figure 10:**
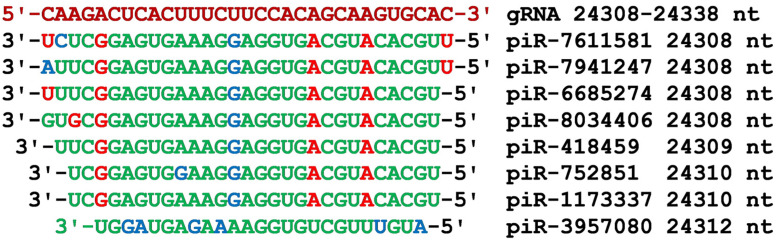
Nucleotide sequences of the seven piRNA BSs cluster in the CDS of gRNA from 24,308 nt to 24,338 nt and the piRNA nucleotide sequences. Note: The piRNA nucleotides that form canonical pairs with gRNA are highlighted in green and non-canonical pairs are highlighted in blue. The piRNA nucleotides that do not interact with gRNA are highlighted in red. The names of the piRNAs are followed by the beginning of their BSs.

The next piRNA BSs cluster from 24,308 nt to 24,338 nt was located in the gRNA region encoding the S surface glycoprotein (Figure 2). All the piRNAs, starting from the 5′-end, contained tetranucleotide CACGU, trinucleotide CGU, pentanucleotide AGGUG, and nanonucleotide GAGUGAAAG, interacting to form only canonical base pairs (Figure 10). These seven piRNAs, ranging in length from 28 nt to 31 nt, bound to gRNA with free energy from −130 kJ/mol to −138 kJ/mol (Table S1).

By replacing nucleotides forming non-canonical pairs with nucleotides forming canonical pairs in piRNAs and replacing non-interacting nucleotides, the resulting spiRNAs bound to gRNA with free energy from −152 kJ/mol to −165 kJ/mol. Such large values of ΔG are also associated with the length of piRNAs from 28 nt to 31 nt. These spiRNAs, fully complementary to gRNAs, are identical in function to widely used siRNAs that cause inhibition of viral RNA protein synthesis [51,52,53]. These examples demonstrate the possibility of creating drugs that suppress the reproduction of coronavirus in human cells.

**Figure 11:**
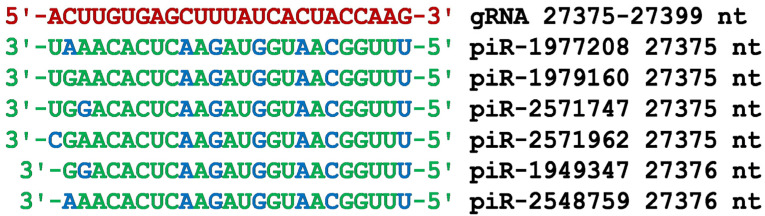
Nucleotide sequences of the six piRNA BSs cluster in the CDS of gRNA from 27,375 nt to 27,399 nt and the nucleotide sequences of piRNAs. Note: The piRNA nucleotides that form canonical pairs with gRNA are highlighted in green and non-canonical pairs are highlighted in blue. The names of the piRNAs are followed by the beginning of their BSs.

The gRNA region from 27,375 nt to 27,399 nt encodes the ORF7a protein (Figure 11). Most of the nucleotides of the identified piRNAs formed canonical pairs, and in general, all nucleotides of these piRNAs formed hydrogen bonds with gRNA nucleotides. All piRNAs had an identical oligonucleotide ACACUCAAGAUGGUAACGGUUU from the 5′ end, which indicates almost complete interchangeability of piRNAs in the putative function of inhibition of ORF7a protein synthesis. piRNAs associated with gRNA in the cluster will interfere with the replication of the coronavirus genome. The localization of the cluster of piRNA BSs from 27,375 nt to 27,399 nt, which is close to the 3′UTR, will terminate early gRNA replication that starts from the 3′UTR.

**Figure 12:**
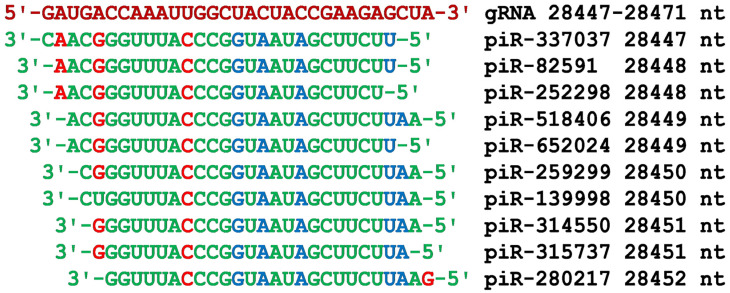
Nucleotide sequences of the 10 piRNA BSs cluster in the CDS of gRNA from 28,447 nt to 28,471 nt and the nucleotide sequences of piRNAs. Note: The piRNA nucleotides that form canonical pairs with gRNA are highlighted in green and non-canonical pairs are highlighted in blue. The piRNA nucleotides that do not interact with gRNA are highlighted in red. The names of the piRNAs are followed by the beginning of their BSs.

In the gRNA region encoding the N protein, a cluster of piRNA BSs from 28,447 nt to 28,471 nt was detected (Figure 2). All piRNAs from the 5′ end, starting from piR-252298, had an identical oligonucleotide CCGGUAAUAGCUUCU, and then the hexanucleotide GGUUUA was located, which bound only through canonical nucleotide pairs (Figure 12). The BSs of piR-806264 and piR-1125646 were located at positions 28,360 nt and 28,636 nt, respectively, from the 5′ end of the cluster and the 3′ end of the cluster (Table S1, see Supplementary Materials). The interaction energy of these 33 nt piRNAs was −142 kJ/mol and −134 kJ/mol, respectively, indicating the importance of these piRNAs in the inhibition of N protein synthesis.

**Figure 13:**
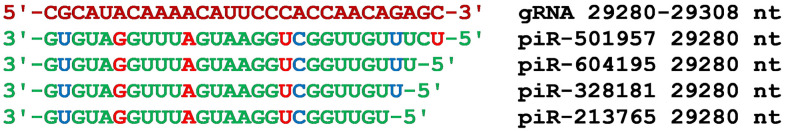
Nucleotide sequences of the 4 piRNA BSs cluster of gRNA from 29,280 nt to 29,308 nt and the nucleotide sequences of piRNAs. Note: The piRNA nucleotides that form canonical pairs with gRNA are highlighted in green and non-canonical pairs are highlighted in blue. The piRNA nucleotides that do not interact with gRNA are highlighted in red. The names of the piRNAs are followed by the beginning of their BSs.

The last cluster of piRNA BSs was located in the region from 29,280 nt to 29,308 nt of gRNA, which also encodes the N protein (Figure 13). Before this BSs cluster at position 29,030 nt was the piR-1134823 BS and after position 29,377 nt was the piR-3158024 BS, both with an interaction energy of −130 kJ/mol (Table S1). That is, as in the previous cluster of BSs, this cluster of four piRNAs BSs is “insured” by the binding of piRNAs before and after the BSs cluster. Four piRNAs contained identical 3′-GUGUAGGUUUAGUAAGGUCGGUUGU-5′ nucleotides, which indicates their almost complete interchangeability in the suppression of N protein synthesis.

**Figure 14:**
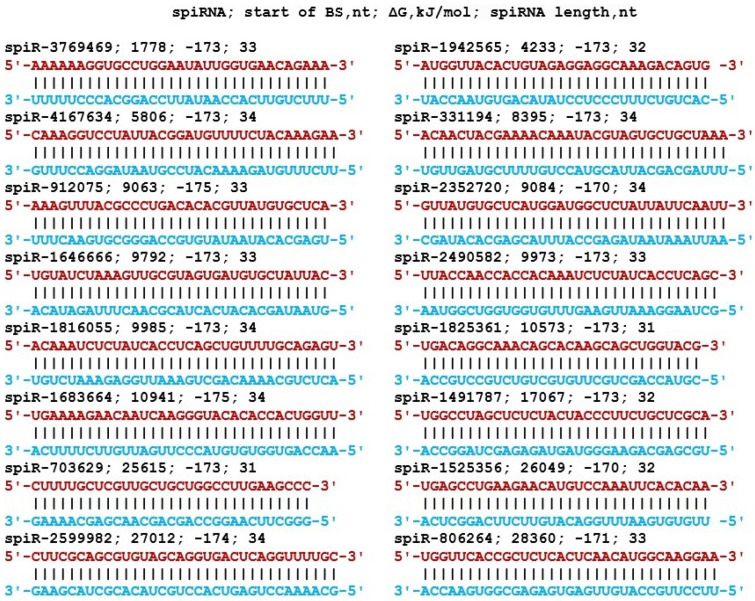
Nucleotide sequences of spiRNAs and characteristics of their interaction in gRNA regions. Note: The gRNA nucleotides of the cluster of spiRNA BSs are highlighted in red. Nucleotides of spiRNAs forming canonical base pairs with gRNA are highlighted in blue.

The clusters of piRNA BSs in gRNA that we have identified form the basis for selecting the most effective piRNAs that can be used to suppress SARS-CoV-2 proliferation. From the list of piRNAs (Table S1) that interacted with gRNA with a free energy of −130 kJ/mol or more, we selected 16 piRNAs based on which, by replacing nucleotides, we made the spiRNA nucleotide sequences that interact with gRNA in completely complementary way by forming canonical nucleotide pairs. The interaction patterns of spiRNAs and gRNA BSs are shown in Figure 14. Each of these spiRNAs will reliably block both the synthesis of the protein encoded by the spiRNAs target and gRNA replication. The effectiveness of spiRNAs is expected to be the same as in many experiments using synthetic interfering siRNA molecules [51,52,53,54,55].

## 4. Discussion

Coronavirus has been found in many animal organisms, which indicates the early occurrence of their protective methods to fight this pathogen [56,57,58]. It is obvious that the low mortality of animal organisms is due to the creation in the process of evolution of endogenous substances that prevent high lethality from coronaviruses. At present, there is information about the involvement of piRNAs in the regulation of the expression of protein-coding genes [45,59]. Since gRNA has features of the mRNA structure, that is, 5′UTR, CDS and 3′UTR, it is logical to assume that the synthesis of proteins based on gRNA as a template can also be regulated by piRNAs. The possibility of the influence of piRNAs on the synthesis of coronavirus proteins has previously been shown [45]. In this work, we show the possibility of piRNAs affecting protein synthesis by binding piRNAs to the gRNA of the Omicron variant of the SARS-CoV-2 genome.

Of the 8,426,000 piRNAs, 92 piRNAs could bind to gRNA with a value of −130 kJ/mol or more (Table S1). These piRNAs bound fully complementarily through the interaction of canonical and non-canonical base pairs. The length of these piRNAs varied from 28 nt to 34 nt, indicating a strong interaction of piRNAs with gRNA. The chosen selection criteria for piRNAs strongly interacting with gRNA made it possible to identify the gRNA regions to which two or more piRNAs bound with overlapping nucleotide sequences of BSs. Such regions, which we called clusters of miRNAs and piRNA BSs, indicate that the corresponding piRNAs can more effectively suppress both protein synthesis on mRNA and gRNA and gRNA replication [60,61,62,63,64,65]. The strong interaction of several piRNAs in the gRNA BSs cluster makes it possible to reliably suppress the reproduction of the coronavirus since a decrease in the concentration of one of the piRNAs will not significantly affect the inhibitory effect of the piRNAs group. Some piRNAs bound to mRNAs of human protein-coding genes, and the diversion of such piRNAs would facilitate the coronavirus replication. Based on this, it is required to control the concentration of these piRNAs in cells and the body to inhibit the reproduction of coronavirus. Logically, the human body produces many piRNAs that can suppress the coronavirus to reliably protect the body from this pathogen. Let us consider the features of the interaction of piRNAs with gRNA to understand why BSs clusters are located in different regions of gRNA. The gRNA of the coronavirus, like protein-coding genes, contains 5′UTR, CDS and 3′UTR (Figure 2). The first cluster of piRNA BSs with gRNA is located in the 5′UTR. Figure 2 shows how piRNAs can interact with gRNA. This localization of the piRNA BSs cluster in the 5′UTR immediately prevents the binding of ribosomes to gRNA and blocks the synthesis of coronavirus proteins. The location of the piRNA BSs cluster allows the use of several spiRNAs based on these piRNAs that will fully complementarily bind in this cluster and will reliably suppress the synthesis of encoded gRNA proteins.

Single nucleotide mutations in BS clusters cannot significantly protect the coronavirus from piRNAs. However, substitutions of three or four nucleotides in the piRNA BSs cluster with a length of 30–34 nucleotides can significantly reduce their impact on coronavirus reproduction. Therefore, during the evolution of animals, they selected longer piRNAs to protect against coronavirus. For this reason, miRNAs, which are much shorter than piRNAs, are less used by animals to protect against coronavirus. The clusters of long piRNA BSs in gRNA that we found are an indicator of the protective function of the body.

In addition to BSs clusters of long piRNAs in gRNA, we found BSs clusters for 24–28 nt long piRNAs. Dozens of piRNAs of this length can bind in these clusters. The best example of this is the cluster of piRNA BSs in the gRNA region from 12,029 nt to 12,055 nt (Figure 7). Such a number of piRNAs is guaranteed to suppress the synthesis of the protein that is involved in the formation of the coronavirus envelope. A similar cluster of piRNA BSs was found in the region from 7137 nt to 7161 nt (Figure 5). This region is included in the nucleotides encoding the nsp3 protein (Figure 2). In this cluster, all nucleotides of 14 piRNAs were involved in the formation of hydrogen bonds with gRNA. In addition to this BSs cluster, two more BSs clusters were found in the nsp3 protein gene (Figure 4 and Figure 6). In the cluster of BSs from 7467 nt to 7492 nt, all the piRNA nucleotides also interact with gRNA (Figure 6). In the second half of the gRNA, in the nucleotide sequence from 20,598 nt to 20,624 nt, there was a large cluster of 18 piRNAs BSs (Figure 9), which can be an effective target for piRNAs. In addition to the clusters of piRNA BSs described above, several more clusters were identified for a smaller number of piRNAs (Figure 2). The results obtained indicate a developed system for protecting the human body from coronavirus using piRNAs. However, not all body cells synthesize the entire set of eight million piRNAs. Therefore, coronaviruses manage to multiply in body cells in which the set of antiviral piRNAs is small or absent. The information on antiviral piRNAs in such cells is crucial since they can be targeted for their protection. Exosomes and vesicles in which miRNAs and piRNAs circulate in the body range in size from 30 to 200 nm [66,67]. In this regard, it is important to understand in which tissues and cells a low level of expression of antiviral piRNAs is found, at least among the most potent piRNAs.

## 5. Conclusions

The data obtained in this work on the effect of piRNAs on coronavirus can be used to predict the likelihood of infection with coronavirus and in the development of drugs based on endogenous piRNAs and spiRNAs. It is logical to create spiRNAs based on piRNAs that are 30–34 nt long that interact with gRNAs with high free energy and bind in the gRNA regions encoding the most important protein components of the coronavirus. At the same time, these highly binding piRNAs will also interfere with gRNA replication.

## Data Availability

Not applicable.

## Supplementary Materials

The following supporting information can be downloaded at: https://www.mdpi.com/article/10.3390/cimb45040193/s1, Table S1: Characteristics of human piRNA interaction with the gRNA of the SARS-CoV-2 omicron strain.
