# Supplementary material for: Endogenous piRNAs Can Interact with the Omicron Variant of the SARS-CoV-2 Genome
Source: Curr Issues Mol Biol. 2023 Apr 3;45(4):2950–64. doi: 10.3390/cimb45040193 (PMC10136802; doi:10.3390/cimb45040193)
Supplement: Supplementary file 1 [file cimb-45-00193-s001.zip › cimb-2227970-supplementary.pdf]

Table S1. Characteristics of human piRNA interaction with gRNA SARS-CoV-2 omicron strain

piRNA	Binding site, nt	Region gRNA	ΔG, kJ/mol	ΔG/ΔGm, %	piRNA length, nt
piR-2388809	156	5'UTR	-132	84	30
piR-7726820	538	CDS	-132	81	31
piR-8103557	641	CDS	-136	81	33
piR-7741905	642	CDS	-130	80	31
piR-3769469	1778	CDS	-138	80	33
piR-3942773	4207	CDS	-138	86	30
piR-4093935	4210	CDS	-136	82	31
piR-1942565	4233	CDS	-138	80	32
piR-4167634	5806	CDS	-140	81	34
piR-331194	8395	CDS	-138	80	34
piR-912075	9063	CDS	-140	80	33
piR-2352720	9084	CDS	-138	81	34
piR-1646666	9792	CDS	-136	80	33
piR-2490582	9973	CDS	-138	80	33
piR-1816055	9985	CDS	-138	80	34
piR-1825361	10573	CDS	-138	80	31
piR-1683664	10941	CDS	-140	80	34
piR-1491787	17067	CDS	-138	80	32
piR-7941247	24308	CDS	-138	84	31
piR-8034406	24308	CDS	-138	84	30
piR-6685274	24308	CDS	-136	85	30
piR-7611581	24308	CDS	-134	81	31
piR-418459	24309	CDS	-136	88	29
piR-752851	24310	CDS	-130	85	28
piR-1173337	24310	CDS	-132	87	28
piR-703629	25615	CDS	-142	82	31
piR-1525356	26049	CDS	-136	80	32
piR-2599982	27012	CDS	-144	82	34
piR-806264	28360	CDS	-142	83	33
piR-1125648	28636	CDS	-134	82	33
piR-1134823	29030	CDS	-130	82	31
piR-3158024	29377	CDS	-130	80	32
